# Alterations of neural network organization during REM sleep in women: implication for sex differences in vulnerability to mood disorders

**DOI:** 10.1186/s13293-020-00297-5

**Published:** 2020-04-25

**Authors:** Matthieu Hein, Jean-Pol Lanquart, Gwénolé Loas, Philippe Hubain, Paul Linkowski

**Affiliations:** Erasme Hospital, Department of Psychiatry and Sleep Laboratory, Université libre de Bruxelles, ULB, Route de Lennik, 808, 1070 Anderlecht, Brussels, Belgium

**Keywords:** Small-world network organization, Sex differences, REM sleep, Slow-wave sleep, Healthy individuals

## Abstract

**Background:**

Sleep plays an important role in vulnerability to mood disorders. However, despite the existence of sex differences in vulnerability to mood disorders, no study has yet investigated the sex effect on sleep network organization and its potential involvement in vulnerability to mood disorders. The aim of our study was to empirically investigate the sex effect on network organization during REM and slow-wave sleep using the effective connectivity measured by Granger causality.

**Methods:**

Polysomnographic data from 44 healthy individuals (28 men and 16 women) recruited prospectively were analysed. To obtain the 19 × 19 connectivity matrix of all possible pairwise combinations of electrodes by Granger causality method from our EEG data, we used the Toolbox MVGC multivariate Granger causality. The computation of the network measures was realized by importing these connectivity matrices into EEGNET Toolbox.

**Results:**

In men and women, all small-world coefficients obtained are compatible with a small-world network organization during REM and slow-wave sleep. However, compared to men, women present greater small-world coefficients during REM sleep as well as for all EEG bands during this sleep stage, which indicates the presence of a small-world network organization less marked during REM sleep as well as for all EEG bands during this sleep stage in women. In addition, in women, these small-world coefficients during REM sleep as well as for all EEG bands during this sleep stage are positively correlated with the presence of subclinical symptoms of depression.

**Conclusions:**

Thus, the highlighting of these sex differences in network organization during REM sleep indicates the presence of differences in the global and local processing of information during sleep between women and men. In addition, this small-world network organization less marked during REM sleep appears to be a marker of vulnerability to mood disorders specific to women, which opens up new perspectives in understanding sex differences in the occurrence of mood disorders.

## Highlights

- Women present small-world network organization less marked during REMS
- There are no sex differences in small-world network organization during SWS
- These results help to better understand sex differences in mood disorders

## Introduction

At the cerebral level, there are several elements in favour of the presence of a small-world network (SWN) organization both during wakefulness and sleep [1–4]. The presence of this SWN cerebral organization seems to promote the emergence of complex behaviours and the optimal cognitive functioning [5, 6] through minimal wiring cost and high dynamic complexity characterized by a rapid transfer and synchronization of information as well as a balance between local processing and global integration [7]. Indeed, the presence of optimal SWN organization is associated with more efficient cognitive functioning [8], whereas the presence of altered SWN organization favours the occurrence of cognitive dysfunctions [9, 10]. Thus, the SWN organization seems to be a very attractive model to explain the organization of brain anatomical, functional or effective networks given its compatibility with both segregated/specialized and distributed/integrated information processing [11].

Sex is a central factor in the inter-individual variations of the human brain [12, 13]. Indeed, in the literature, there are many arguments in favour of a cerebral sex dimorphism at the anatomical, morphological, metabolic, neurochemical and neurophysiologic level [12, 13]. In addition, this cerebral sex dimorphism is also characterized by the presence of specific patterns of SWN organization in men and women during wakefulness, which seems to indicate the existence of sex differences in the cerebral processing of information [14–20]. Nevertheless, this potential effect of sex on SWN organization has not yet been investigated during sleep. However, it has been shown that sex may strongly influence both the cognitive functions related to sleep (such as memory and regulation of emotions) [21, 22] and the sleep architecture [23]. Indeed, compared to men, women present a specific polysomnographic pattern characterized by modifications of slow-wave sleep and disinhibition of rapid eye movement (REM) sleep [24, 25]. Moreover, these particular polysomnographic characteristics have been identified as markers of vulnerability in individuals at high risk of mood disorders [26], which may be explained by the implication of these sleep stages in the pathophysiology of mood disorders [27–30]. However, women seem to have a higher vulnerability to mood disorders [31] that could be partially induced by this sex dimorphism in these sleep stages [32]. Thus, given the presence of this sleep sex dimorphism and its potential pathophysiological implications for mood disorders, it seems important to study the potential effect of sex on SWN organization during sleep in order to highlight possible sex differences in the cerebral processing of information during sleep and their possible implications in the vulnerability to mood disorders.

Our first hypothesis was that there were sex differences in SWN organization during REM and slow-wave sleep. Our second hypothesis was that these sex differences in SWN organization during these sleep stages were correlated with the presence of subclinical symptoms of depression. In order to test these hypotheses, we investigated empirically the sex effect on SWN organization during REM and slow-wave sleep as well as for all EEG bands during these sleep stages using the effective connectivity measured by the Granger causality in healthy individuals. The aim of our study was to highlight sex differences in the global and local processing of information during these sleep stages in order to better understand the differences in the occurrence of mood disorders between men and women.

## Material and methods

The methodology used in this study is similar to that used in previous studies of our research group on sleep network organization [4, 33].

### Introduction to graph theory analysis

Networks are constituted by a set of nodes (vertices) and links (edges) between the pairs of nodes allowing a mathematical representation of complex biological, social and informatics systems [34]. In addition, each network is characterized by two fundamental parameters: the characteristic path length corresponding to the minimum number of edges needed to make a connection between nodes and the clustering coefficient corresponding to a measure of topological clustering of edges between nodes [35, 36]. The combination of these two parameters leads to the determination of three types of networks according to their characteristics: ordered (characterized by a high clustering coefficient and long path length), random (characterized by a low clustering coefficient and short path length) and small-world (characterized by a high clustering coefficient and short path length) [37].

In order to study these particular network organizations, it is possible to use several methods based on different types of connectivity: anatomical (based on the physical or structural connections between the various brain structures), functional (measuring the statistical dependence between distant cerebral structures and highly time-dependent) and effective (measuring the direct or indirect causal influences between two brain regions) [38, 39]. Among the methods to study the effective connectivity, the Granger causality is an application of time series based on the following prediction: “If the predicted error on the first time series is reduced by including measurements of the second in a linear regression model, then the second temporal series has a causal influence on the first” [40, 41]. In addition, it is possible to apply the Granger causality to electrophysiological time series since this type of data may be sampled in a timely manner and there is no lag between recorded responses and their underlying causes [42].

### Population

Through advertisements, we recruited prospectively 44 healthy individuals (28 men and 16 women) between January 2007 and January 2012 from the community. Unlike women, all men included in this study come from our previous studies on sleep network organization [4, 33].

Upon admission to the Sleep Laboratory of the Erasme Hospital, all these individuals benefited:

- From a systematic clinical interview by a unit psychiatrist in order to exclude an axis I or axis II disorder according to the diagnostic criteria of the Diagnostic and Statistical Manual of Mental Disorders Fourth Edition-Text Revision [43].
- From a complete somatic check-up (physical examination, chest X-ray, electrocardiogram (ECG), electroencephalogram (EEG) and laboratory tests, such as blood test and urinalysis) by an internist in order to exclude the presence of somatic conditions or pathologies that may affect sleep.
- From a complete assessment of sleep (systematic sleep-specific interview and sleep examination) by a specialist in sleep medicine in order to exclude the presence of sleep disorders according to the diagnostic criteria of American Academy of Sleep Medicine (such as insomnia disorder, circadian rhythm disorder, obstructive or central sleep apnoea syndrome, restless legs syndrome, periodic limb movements during sleep, parasomnia and hypersomnia disorder) [44], antecedents of sleep disorders and irregular sleep-wake schedules.
- From an assessment of their subjective complaints of depressive symptoms via the Beck Depression Inventory (BDI reduced to 13 items). This scale consists of 13 items that can be scored from 1 to 3. The final score can vary from 0 to 39. A final score of 0–4 indicates an absence of depression, 5–7 a subclinical depression, 8–15 a moderate depression and > 16 severe depression [45].

Moreover, these individuals have never been under somatic or psychotropic treatment (such as antidepressants, thymostabilizing treatments [lithium or anti-epileptic treatments], benzodiazepines, Z-drugs, neuroleptics, antihistamines, opioids, melatonin, plants with psychotropic effect [valerian, passiflore, St. John’s wort, etc.] and psychostimulant treatments) that may influence sleep.

### Methods

**Fig. 1:**
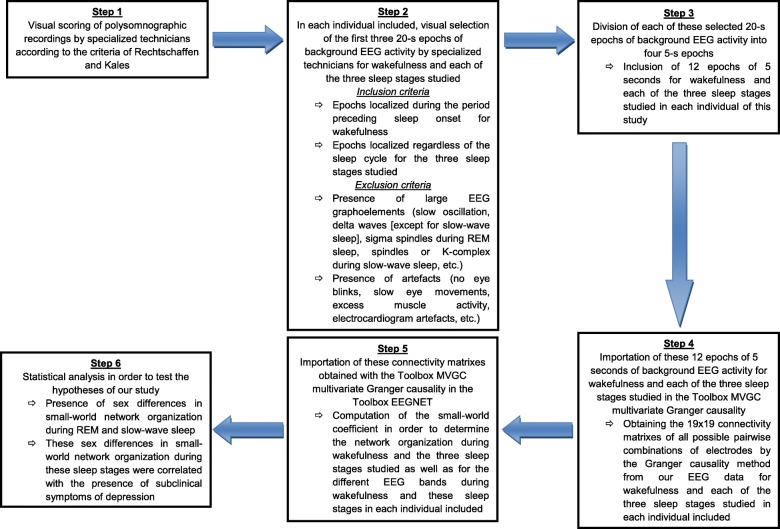
Summary of main steps of the methodology applied in this study for both EEG signal and network analysis

In order to allow the repeatability of our study, the main steps of our methodology are summarized in Fig. 1.

#### EEG recordings and experimental conditions

All individuals included in our study performed three consecutive nights of polysomnographic recording within the Sleep Laboratory of the Erasme Hospital. For each of these nights, the recording time was a minimum of 8 h. Regarding the women included in this study, these three consecutive nights of polysomnographic recording were performed during the follicular phase (outside of menstruation period) of their menstrual cycle in order to avoid potential sleep disturbances associated with post-ovulation luteal phase and menstrual period [46]. To achieve our analysis, we selected an “artefact-free night” from the last two nights recorded in order to avoid the “first night effect” on sleep parameters [47]. However, if these two nights had similar levels of artefacts, the selected night was randomly chosen. To allow the visual detection of these eventual artefacts, our specialized technicians have used the software Endymion (Endymion 1993–2020), Sleep laboratory, Erasme Hospital [48–51] developed in our sleep laboratory for data analysis.

Furthermore, the applied polysomnography-montage, the sampling frequencies of the different channels, the applied analogue filters, the used data format and the instructions to follow during the stay within the Sleep Laboratory are available in the Supplementary data.

In order to determine the different sleep stages, specialized technicians visually scored the polysomnographic recordings according to the criteria of Rechtschaffen and Kales [52]. All subsequent analyses, such as stage determination and spectrum calculation, were carried out on the sampled data, avoiding synchronization problems between the stages and the other calculations.

#### EEG signal analysis

For our analysis, we selected the first three 20-s epochs of background EEG activity without large EEG graphoelements (slow oscillation, delta waves [except for slow-wave sleep], sigma spindles during REM sleep, spindles or K-complex during slow-wave sleep, etc.) and artefacts (no eye blinks, slow eye movements, excess muscle activity, electrocardiogram artefacts, etc.) for wakefulness (during the period preceding sleep onset) and each of the three sleep stages studied (regardless of the sleep cycle). Subsequently, each of these 20-s epochs of background EEG activity has been divided into four 5-s epochs [4, 33]. Thus, for each individual of this study, we have included 12 epochs of 5 s of background EEG activity for wakefulness and each of the three sleep stages studied (REM sleep, stage 3 and stage 4). This number of 20-s epochs (three) used in this study was arbitrarily chosen in order to be able to investigate the network organization of wakefulness and each sleep stage over an identical number of epochs. Indeed, in some individuals included in this study, it was impossible to obtain more than three 20-s epochs of background EEG activity without large EEG graphoelements and artefacts for wakefulness and/or some sleep stages. Furthermore, for wakefulness and each of the three sleep stages studied, the spectral power content of each of these epochs has been calculated in order to check the homogeneity of the epochs included for a given stage (wakefulness, REM sleep, stage 3 and stage 4) in each individual. Finally, given the impossibility of obtaining polysomnographic recordings without any artefacts, this selection of a limited number of epochs of background EEG activity without artefacts for wakefulness and each sleep stage seems to be the best compromise for a quality study of network organization during wakefulness and sleep stages since this approach has already been used by our research group as well as in the literature [2, 4, 33, 53–58].

In our study, we did not investigate the network organization during stage 1 and stage 2 because our aim was to focus on the network organization of sleep stages who are most involved in vulnerability to mood disorders (REM and slow-wave sleep) [59–61].

#### The Granger causality

To obtain the 19 × 19 connectivity matrix of all possible pairwise combinations of electrodes by the Granger causality method from our EEG data, we used the Toolbox MVGC multivariate Granger causality [46] developed for use under Matlab. This toolbox is based on a multivariable autoregressive (MVAR) model of the data. The mathematical developments used in the Toolbox MVGC multivariate Granger causality (model order estimation, MVAR model estimation, time domain analysis, and frequency domain analysis) [62] are beyond the scope of this paper but are summarized in the Supplementary data.

Since both time and frequency domain causalities were obtained, we also calculated the time-domain causalities by integrating their spectral counterparts and checked that this frequency integrated value was not different from time domain value. The frequency-based connectivity matrices were calculated for the following EEG bands: *β* (16–32 Hz), *σ* (12–16 Hz), *α* (8–12 Hz), *θ* (3–8 Hz) and *δ* (0.25–3 Hz) [63]. A comparison of our method based on the Granger causality with other methods in the literature is available in the Supplementary Data.

**Fig. 2:**
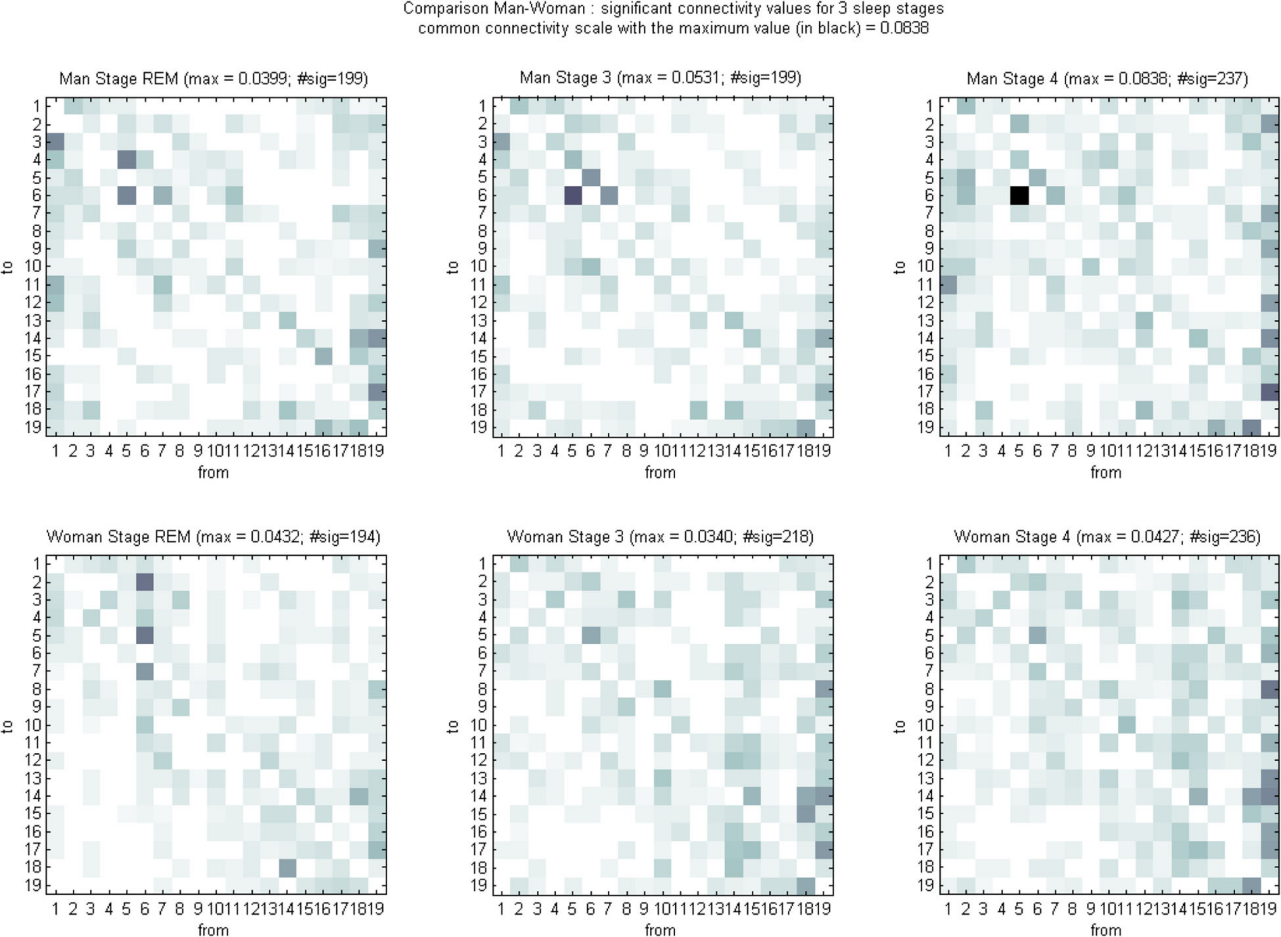
Example of connectivity matrix obtained with the Toolbox MVGC multivariate Granger causality. Legend: 1 = ‘Fp1’, 2 = ‘Fp2’, 3 = ‘F7’, 4 = ‘F3’, 5 = ‘Fz’, 6 = ‘F4’, 7 = ‘F8’, 8 = ‘T3’, 9 = ‘C3’, 10 = ‘Cz’, 11 = ‘C4’, 12 = ‘T4’, 13 = ‘T5’, 14 = ‘P3’, 15 = ‘Pz’, 16 = ‘P4’,17 = ‘T6’, 18 = ‘O1’, 19 = ‘O2’

An example of the connectivity matrix obtained with the Toolbox MVGC multivariate Granger causality [62] for the different sleep stages (REM sleep, stage 3 and stage 4) in a man and a woman from our sample is available (Fig. 2).

#### Calculation of network characteristics

In order to determine the network organization during wakefulness and the three sleep stages studied (stage 3, stage 4 and REM sleep) as well as for the different EEG bands during wakefulness and these sleep stages, we imported the connectivity matrix obtained with the Toolbox MVGC multivariate Granger causality into the Toolbox EEGNET developed for use under Matlab [64]. Indeed, based on graph theory analysis, this software allows the computation of the small-world coefficient (SWC) measuring the propensity of the network to have a small-world structure. Values of SWC are restricted to the interval − 1 to 1 regardless of network size. Values close to zero, positive values and negative values indicate a graph with more small-world, random and regular characteristics, respectively [65].

### Statistical analysis

Statistical analyses were performed using Stata version 14. The normal distribution of the data was verified using histograms, boxplots, and quantile-quantile plots. Since most data followed an asymmetric distribution, we used non-parametric tests for all variables, beginning with the Wilcoxon test to evaluate for significant differences between the medians observed in men and women groups. Results were considered significant when the *p* value was < 0.05 for global tests and < 0.05 after Bonferroni correction for multiple comparisons. Correlation analyses were performed using Spearman correlations.

## Results

### Demographic and polysomnographic data (Table 1)

Compared to men, women showed greater BDI scores. However, despite this significant difference in the BDI, scores remained below 8 for both men and women, indicating the absence of clinical depression. There were no significant sex differences for age, educational level, social status and possible consumption (caffeine, tobacco and alcohol).

**Table 1:** Comparison of demographic and polysomnographic data between men and women groups

	Median (P25–P75) Men group (*N* = 28)	Median (P25–P75) Women group (*N* = 16)	*P* value
**Demographic variables**			
Age (years)	25 (22–34.5)	22.5 (21.5–25)	0.157
BDI score	1 (0–3)	4 (3–6)	< 0.001
Social status			0.336
Unmarried	60.7%	75.0%	
Married	39.3%	25.0%	
Educational level			0.165
University students	46.4%	75.0%	
University graduates	21.4%	6.3%	
Non-university graduates	23.1%	18.7%	
Smoking			0.552
No	92.9%	87.5%	
Yes	7.1%	12.5%	
Alcohol			0.832
No	78.6%	81.2%	
Occasional	21.4%	18.8%	
Caffeine			0.447
No	42.9%	31.2%	
Yes	57.1%	68.8%	
**Polysomnographic variables**			
SL (min)	20.83 (13.67–31.50)	34.34 (19.67–61.00)	0.038
SE (%)	87.33 (84.18–91.65)	87.12 (83.03–93.34)	0.961
SPT (min)	536.50 (509.33–564.00)	510.84 (499.17–518.67)	0.031
TST (min)	493.67 (466.67–515.50)	476.00 (452.17–497.67)	0.113
WASO (%)	6.87 (5.00–11.75)	5.41 (2.72–11.17)	0.223
Stage 1 (%)	6.90 (5.76–8.96)	5.35 (4.21–6.68)	0.017
Stage 2 (%)	51.70 (47.07–55.29)	45.89 (41.84–53.90)	0.118
Stage 3 (%)	6.06 (5.01–7.06)	7.02 (5.24–7.65)	0.278
Stage 4 (%)	7.27 (4.23–11.20)	10.91 (5.87–13.06)	0.134
SWS (%)	14.43 (10.32–16.99)	16.93 (12.73–20.98)	0.140
REM (%)	20.04 (17.75–21.71)	22.19 (20.37–24.29)	0.036
REM latency (min)	66.50 (58.17–77.17)	52.50 (49.17–63.83)	0.047

*BDI* Beck depression inventory, *SL* Sleep latency, *SE* Sleep efficiency, *SPT* Sleep period time, *TST* Total sleep time, *REM* Rapid eye movement sleep, *WASO* wake after sleep onset.

### Polysomnographic data (Table 1)

Compared to men, women had greater sleep latency and percentage of REM sleep, smaller sleep period time and percentage of stage 1 and shorter REM latency. There were no significant sex differences for other polysomnographic parameters.

### Network parameters—time domain (Table 2)

Compared to men, women showed greater SWC during REM sleep. There were no significant sex differences at the level of SWC for stage 3, stage 4 and wakefulness.

**Table 2:** Comparison of network characteristics between men and women groups

	Median (P25–P75) Men group (*N* = 28)	Median (P25–P75) Women group (*N* = 16)	*P* value
**Wakefulness—Time domain**			
SWC	− 0.002 (− 0.005–0.014)	0.010 (0.002–0.035)	0.060
**Wakefulness - Frequency domain**			
SWC—power bands β	− 0.002 (− 0.004–0.004)	− 0.001 (− 0.010–0.038)	0.558
SWC—power bands σ	− 0.006 (− 0.011–0.008)	− 0.002 (− 0.013–0.036)	0.591
SWC—power bands α	− 0.005 (− 0.011–0.010)	− 0.005 (− 0.020–0.043)	0.961
SWC—power bands θ	− 0.004 (− 0.010–0.012)	0.008 (− 0.009–0.033)	0.196
SWC—power bands δ	− 0.004 (− 0.011–0.011)	− 0.003 (− 0.014–0.033)	0.661
**REM—time domain**			
SWC	0.054 (0.035–0.091)	0.094 (0.060–0.147)	0.007
**REM—frequency domain**			
SWC—power bands β	0.075 (0.042–0.110)	0.122 (0.083–0.174)	0.006
SWC—power bands σ	0.068 (0.031–0.110)	0.120 (0.084–0.156)	0.005
SWC—power bands α	0.076 (0.042–0.109)	0.133 (0.077–0.172)	0.011
SWC—power bands θ	0.065 (0.032–0.120)	0.115 (0.075–0.173)	0.006
SWC—power bands δ	0.068 (0.049–0.103)	0.124 (0.082–0.172)	0.007
**Stage 3—time domain**			
SWC	0.009 (0.0004–0.033)	0.036 (0.007–0.061)	0.242
**Stage 3—frequency domain**			
SWC—power bands β	0.013 (− 0.001–0.043)	0.042 (0.001–0.064)	0.341
SWC—power bands σ	0.011 (− 0.003–0.046)	0.028 (− 0.006–0.065)	0.733
SWC—power bands α	0.002 (− 0.004–0.028)	0.018 (− 0.007–0.061)	0.526
SWC—power bands θ	0.011 (− 0.007–0.035)	0.038 (− 0.003–0.071)	0.575
SWC—power bands δ	0.013 (− 0.004–0.061)	0.047 (0.010–0.071)	0.150
**Stage 4—time domain**			
SWC	0.006 (− 0.003–0.034)	0.022 (− 0.004–0.044)	0.714
**Stage 4—frequency domain**			
SWC—power bands β	0.003 (− 0.007–0.039)	0.015 (− 0.009–0.045)	0.714
SWC—power bands σ	0.005 (− 0.006–0.042)	0.019 (− 0.009–0.052)	0.678
SWC—power bands α	0.004 (− 0.006–0.044)	0.012 (− 0.009–0.050)	0.788
SWC—power bands θ	− 0.001 (− 0.007–0.028)	0.012 (− 0.013–0.039)	0.845
SWC—power bands δ	0.002 (− 0.005–0.036)	0.020 (− 0.010–0.047)	0.558

*REM* rapid eye movement sleep, *SWC* small world coefficient

### Network parameters—frequency domain (Table 2)

For all EEG bands during REM sleep, women have greater SWC compared to men. There were no significant sex differences at the level of SWC for all EEG bands during stage 3, stage 4 and wakefulness.

### Correlations analyses

#### Correlations between sex and network parameters (Table 3)

Woman sex was positively correlated with SWC only for REM sleep as well as for all EEG bands during this sleep stage. There were no significant correlations between woman sex and SWC for stage 3, stage 4 and wakefulness.

**Table 3:** Correlations between sex and network parameters

(*N* = 44)	Small world coefficient						
**Woman sex**							
**REM**	0.409^a^	**Stage 3**	0.179	**Stage 4**	0.056	**Wakefulness**	0.287
Power band β	0.417^a^	Power band β	0.145	Power band β	0.056	Power band β	0.089
Power band σ	0.432^a^	Power band σ	0.052	Power band σ	0.063	Power band σ	0.082
Power band α	0.387^a^	Power band α	0.097	Power band α	0.041	Power band α	0.007
Power band θ	0.417^a^	Power band θ	0.086	Power band θ	0.030	Power band θ	0.197
Power band δ	0.413^a^	Power band δ	0.222	Power band δ	0.089	Power band δ	0.067
							^a^*p* < 0.05

*REM* rapid eye movement sleep

^a^ indicates the presence of a significant correlation (*p* <0.05)

#### Correlations between BDI scores and network parameters (Table 4)

In women, BDI scores were positively correlated with SWC for REM sleep as well as for all EEG bands during this sleep stage, whereas in men, there were no significant correlations between BDI scores and SWC for this sleep stage. Both in men and women, the other correlations analyses between BDI scores and SWC were not significant for stage 3, stage 4 and wakefulness.

**Table 4:** Correlations between BDI scores and network parameters

	Small world coefficient						
**BDI score**							
**Men sex (*n*= 28)**							
REM	0.031	Stage 3	0.212	Stage 4	0.064	Wakefulness	− 0.167
Power band β	0.061	Power band β	0.259	Power band β	− 0.154	Power band β	− 0.076
Power band σ	0.135	Power band σ	0.239	Power band σ	0.062	Power band σ	− 0.141
Power band α	0.072	Power band α	0.110	Power band α	− 0.089	Power band α	− 0.047
Power band θ	− 0.083	Power band θ	0.095	Power band θ	− 0.023	Power band θ	− 0.341
Power band δ	0.032	Power band δ	0.200	Power band δ	− 0.017	Power band δ	− 0.301
**Woman sex (*n*= 16)**							
REM	0.636^a^	Stage 3	0.167	Stage 4	− 0.134	Wakefulness	− 0.009
Power band β	0.593^a^	Power band β	0.070	Power band β	− 0.111	Power band β	0.158
Power band σ	0.643^a^	Power band σ	− 0.003	Power band σ	0.006	Power band σ	− 0.214
Power band α	0.627^a^	Power band α	0.085	Power band α	− 0.088	Power band α	− 0.087
Power band θ	0.612^a^	Power band θ	− 0.097	Power band θ	− 0.197	Power band θ	0.137
Power band δ	0.608^a^	Power band δ	0.130	Power band δ	− 0.046	Power band δ	0.251
							^a^*p* < 0.05

*BDI* Beck depression inventory, *REM* rapid eye movement sleep

^a^ indicates the presence of a significant correlation (*p* <0.05)

### Comparisons of network parameters between the different sleep stages and wakefulness (Supplementary data—Table 1)

Both in men and women, REM sleep showed greater SWC than stage 3, stage 4 and wakefulness. All other comparisons between the different sleep stages and wakefulness were no significant.

## Discussion

In our study, we have shown that all SWC obtained are compatible with a SWN organization during REM and slow-wave sleep for both men and women. However, compared to men, women present greater SWC during REM sleep as well as for all EEG bands during this sleep stage, which means that the SWC values during REM sleep as well as for all EEG bands during this sleep stage deviate more from the small-world characteristic threshold in women than in men. In addition, in women, SWC during REM sleep as well as for all EEG bands during this sleep stage seem to deviate more from the values of wakefulness than in men. These different elements therefore indicate the presence of a SWN organization less marked during REM sleep as well as for all EEG bands during this sleep in women. Moreover, woman sex is positively correlated with SWC only for REM sleep as well as for all EEG bands during this sleep stage. Finally, in women, these SWC during REM sleep as well as for all EEG bands during this sleep stage are positively correlated with the presence of subclinical symptoms of depression.

In the literature, there are several elements in favour of a modulatory effect of sex steroid hormones on some structural and functional brain connectivity parameters such as white matter structure, grey matter structure and overall network connectivity [66]. Indeed, at the cerebral level, ovarian hormones (oestrogen and progesterone) seem to favour both cortico-cortical and subcortico-cortical functional connectivity, whereas androgens (testosterone) appear to decrease subcortico-cortical functional connectivity and increase functional connectivity between subcortical brain structures [67]. In addition, these sex steroid hormones play an important role in overall functional connectivity of cerebral hemispheres by promoting intra-hemispheric functional connectivity in men and inter-hemispheric functional connectivity in women [68]. This modulatory effect of sex steroid hormones on cerebral connectivity may allow a better understanding of sex differences highlighted in our study at the sleep network organization level. Indeed, given the existence during REM sleep of a desynchronized cortical EEG activity and a rupture of the communication between the anterior and posterior cortical areas [69–71], the presence in men of a better connectivity between subcortical brain structures [67] playing an important role in REM sleep regulation [72] and a better intra-hemispheric connectivity promoting more efficient antero-posterior communication within the cerebral hemispheres [68] may explain the presence in men of a SWN organization more marked during REM sleep as well as for all EEG bands during this sleep stage compared to women. On the other hand, the lack of sex differences in the SWN organization during slow-wave sleep may be explained by the fact that slow-wave sleep is characterized by the presence of highly synchronized delta waves associated with diffuse communication between different cerebral areas regardless of sex [69–71]. Thus, there is a sex effect on the sleep network organization only during REM sleep as well as for all EEG bands during this sleep stage, which seems to indicate the presence of sex differences in the local and global processing of information during this sleep stage.

Unlike the literature [14–20], we did not highlight any sex differences in SWN organization during wakefulness, which could be explained by the fact that in this study, we selected only epochs of wakefulness during the period preceding sleep onset. Indeed, during this particular period of wakefulness, there is a gradual decrease in the high-frequency EEG bands (*β* and *α*) and a gradual increase in the low-frequency EEG bands (*δ*) in order to prepare the sleep-wake transition [73]. However, the presence of these modifications in the frequency bands during this period preceding sleep onset seems to be associated with a more efficient antero-posterior cerebral communication favouring a more marked SWN organization in both men and women [74]. Furthermore, we demonstrated that compared to men, women had a greater sleep latency and a shorter sleep period time which seems to indicate the presence of reduced sleep duration in women. However, one of the possible compensatory mechanisms in case of reduced sleep duration is the presence of a more marked SWN organization during the period of wakefulness following this reduced sleep duration [75]. Thus, the presence of this reduced sleep duration in women compared to men could possibly explain the sex differences in SWN organization during wakefulness highlighted in the literature [18, 19].

The presence of a positive correlation between SWC during REM sleep as well as for all EEG bands during this sleep stage and subclinical symptoms of depression in women could allow a better understanding of sex differences in vulnerability to mood disorders [76]. Indeed, in major depression, there is a deregulation of REM sleep (characterized by REMS increased, REM latency shortened and REM density increased) [77] secondary to alterations in neurotransmission characterized by hypoactivity of the monoaminergic system and hyperactivity of the cholinergic system [78]. Moreover, in major depressed individuals, these alterations of neurotransmission related to REM sleep lead to changes in both processing and transmission pathways of information [79], which may induce a SWN organization less marked during REM sleep [2, 33]. However, these different alterations of REM sleep induce and maintain cognitive distortions playing a central role in the pathophysiology of major depression [80]. Nevertheless, similar to major depression [33], women (compared to men) present a SWN organization less marked during REM sleep as well as a particular pattern of REM sleep (characterized by REMS increased and REM latency shortened) [24, 25] probably induced by the action of ovarian hormones on the neurotransmission pathways involved in the REM sleep regulation [81]. Thus, the presence of these particular features of REM sleep in women could predispose them to develop subclinical symptoms of depression (such as cognitive distortions [including negative self-esteem and the overnight consolidation of negatively toned emotional memories]) favouring the emergence of mood disorders [80].

### Limitations

In our study, we measured the effective connectivity determined by the Granger causality only at the level of the scalp, which may limit the interpretations of our results. Otherwise, although we only analysed data from a relatively small group of healthy individuals, the samples included in this study were at least similar or even larger than those of most other studies investigating the SWN organization during sleep [2, 4, 33, 55, 57, 69, 75, 82–87], which should allow an adequate interpretation of our results. However, in order to confirm the results highlighted in our study, it seems important to carry out replication studies on samples at least similar to those in our study. Moreover, in our study, we included only non-menopausal women in the follicular phase (outside of menstruation period) of their menstrual cycle, which mean that our results cannot be generalized to women in the post-ovulation luteal phase of their menstrual cycle or menopausal. Currently, there are several methods based on the effective connectivity measured by the Granger causality (each having their advantages and disadvantages) developed by different research teams, which leads to many expert debates on the preferential method [88].

### Perspectives and significance

Despite its limitations, this paper confirms the existence of a sex difference in sleep network organization, which had not yet been demonstrated in the literature. Indeed, compared to men, women present greater small-world coefficients during REM sleep as well as for all EEG bands during this sleep stage, which indicates the presence of a small-world network organization less marked during REM sleep as well as for all EEG bands during this sleep in women. Moreover, in women, these small-world coefficients during REM sleep as well as for all EEG bands during this sleep stage are positively correlated with the presence of subclinical symptoms of depression. Thus, the highlighting of these sex differences in network organization during REM sleep indicates the presence of differences in the global and local processing of information during sleep between women and men. In addition, this SWN organization less marked during REM sleep appears to be a marker of vulnerability to mood disorders specific to women, which opens up new perspectives in understanding sex differences in the occurrence of mood disorders.

## Conclusion

In our study, we demonstrated the presence of sex differences in the global and local processing of information during sleep, which could allow a better understanding of differences in vulnerability to mood disorders between men and women.

## Supplementary information

**Additional file 1.** The applied polysomnography-montage, the sampling frequencies of the different channels, the applied analogue filters, the used data format and the instructions to follow during the stay within the Sleep Laboratory.

## Data Availability

The datasets used and/or analysed during the current study are available from the corresponding author on reasonable request.

## Abbreviations

BDI: Beck Depression Inventory
ECG: Electrocardiogram
EEG: Electroencephalogram
EMG: Electromyogram
EOG: Electro-oculogram
MVAR: Multivariable autoregressive
REM: Rapid eye movement
SWC: Small-world coefficients
SWN: Small-world network
