# Supplementary material for: Alterations of neural network organization during REM sleep in women: implication for sex differences in vulnerability to mood disorders
Source: Biol Sex Differ. 2020 Apr 25;11:22. doi: 10.1186/s13293-020-00297-5 (PMC7183628; doi:10.1186/s13293-020-00297-5)
Supplement: Supplementary file 1 — Additional file 1. The applied polysomnography-montage, the sampling frequencies of the different channels, the applied analogue filters, the used data format and the instructions to follow during the stay within the Sleep Laboratory. [file 13293_2020_297_MOESM1_ESM.docx]

**EEG Recordings and Experimental Conditions**

Individuals included in study were instructed to respect their usual sleeping and waking hours, wake up spontaneously in the morning, turn off the lights during the hours spent in bed, refrain from consuming substances that may affect sleep (such as alcohol, coffee or over-the-counter sleep aids) and avoid daytime naps during their stay at the sleep laboratory.

The applied polysomnography-montage (Alice5 TM, Respironics, Murrysville, PA) was as follows: 19 EEG channels placed according to the international 10–20 standard system with a contralateral reference to the A1 or A2 mastoid derivation, two electro-oculogram (EOG) channels, one submental electromyogram (EMG) channel, ECG, thermistors to detect the oro-nasal airflow (InfinityTM, Sleepmate Technologies, Midlothian, VA), finger pulse-oximetry (Masimo MS-7, Irvine CA), a microphone to record breathing sounds and snoring, piezoelectric sensors to measure thoracic and abdominal breathing (Resp-EZTM, Sleepmate Technologies) and ankle piezoelectric movement strain gauges to detect leg movements (Moving ImagesTM, Sleepmate Technologies).

Since our polysomnographic recordings were scored according to the Rechtschaffen and Kales classification, we decided to study stage 3 and stage 4 separately to highlight possible gender differences in their network organization. However, if no gender differences are found in our analyses for stage 3 and stage 4, we will refer to these sleep stages as slow-wave sleep to simplify our discussion.

All channels were sampled at 2,000 Hz—16 bits, whereas for subsequent analyses, the EEG was stored at 200 Hz, the EOG at 100 Hz, the EMG at 100 Hz and the ECG at 500 Hz. Before down sampling, adequate analogue filters were applied to eliminate low frequency artefacts, drifts, offsets and aliasing. The data were exported to the EDF format using Alice Sleepware (Respironics).

**Mathematical developments used in the Toolbox MVGC multivariate Granger causality**

1. Model order estimation: For each model order p up to a chosen maximum, a MVAR model is fit to the full “universe of data” time series and the best model order p according to the criterion derived from information theory is selected.
2. MVAR model estimation: The corresponding MVAR model parameters for the selected model order is estimated and the resulting model is checked for stability (other statistical tests on the MVAR parameters and residuals may be performed at this stage).
3. Time domain: The time-domain conditional Granger causalities are calculated for all pairs of signals. Conditional means that the effects of the others signals are taken into account to avoid possible indirect causal influences. The significance of the resulting causalities is tested, taking care to adjust for multiple hypotheses.
4. Frequency domain: The frequency conditional spectral causalities are calculated for all pairs of signals. The significance of the resulting causalities is tested.

**Comparison of our method based on the Granger causality with the other available methods in the literature**

Unlike other studies available in the literature using the functional connectivity determined by computing the synchronization likelihood to investigate the sleep network organization [1,2,3,4], we decided to use the effective connectivity measured by the Granger causality in our study. Indeed, the effective connectivity analyses allows to highlight the simplest possible circuit diagram explaining observed responses and measure mostly direct or indirect causal influences between two brain regions, which does not allow the functional connectivity analyzes [5]. In addition, unlike the synchronization likelihood, the Granger causality is not limited to measuring a statistical interdependencies between a time series and one or more other time series within a dynamical system since it also allows the identification of directed functional interactions from time series data and makes it possible to add a statistical predictive notion of causality [6,7,8]. Thus, it is possible to identify a directed causal interaction within coupling using the methods based on effective connectivity measured by the Granger causality.

Furthermore, in our study, we decided not to use thresholding techniques [9,10] because we had already selected only the significant connections within the different networks studied via the statistical inference tests of the MVGC Multivariate Granger causality Toolbox [11] and calculated the different network measures via the mathematical methods based on graph theory analysis of the Toolbox EEGNET [12]. However, it may be interesting in the future to conduct studies comparing our methodology and topological thresholding techniques to evaluate the reproducibility of our results.

**Additional references**

# [1]Leistedt SJ, Coumans N, Dumont M, Lanquart JP, Stam CJ, Linkowski P. Altered sleep brain functional connectivity in acutely depressed patients. Hum Brain Mapp. 2009;30(7):2207-2219.

[2]Ferri R, Rundo F, Bruni O, Terzano MG, Stam CJ. Small-world network organization of functional connectivity of EEG slow-wave activity during sleep. Clin Neurophysiol. 2007;118(2):449-456.

[3]Ferri R, Rundo F, Bruni O, Terzano MG, Stam CJ. The functional connectivity of different EEG bands moves towards small-world network organization during sleep. ClinNeurophysiol. 2008;119(9):2026-2036.

[4]Dimitriadis SI, Laskaris NA, Del Rio-Portilla Y, Koudounis G Ch. Characterizing dynamic functional connectivity across sleep stages from EEG. Brain Topogr. 2009;22(2):119-133.

[5]Friston K, Moran R, Seth AK. Analysing connectivity with Granger causality and dynamic causal modelling. Curr Opin Neurobiol. 2013;23(2):172-178.

[6]Stam CJ, van Dijk BW. Synchronization likelihood: An unbiased measure on generalized synchronization in multivariate datasets. Physica D. 2002;19:562–574.

[7]Rosales F, García-Dopico A, Bajo R, Nevado Á. An efficient implementation of the synchronization likelihood algorithm for functional connectivity. Neuroinformatics. 2015;13(2):245-258.

# [8]Seth AK, Barrett AB, Barnett L. Granger causality analysis in neuroscience and neuroimaging. J Neurosci. 2015;35(8):3293-3297.

# [9]Dimitriadis SI, Laskaris NA, Tsirka V, Vourkas M, Micheloyannis S, Fotopoulos S. Tracking brain dynamics via time-dependent network analysis. J Neurosci Methods. 2010;193(1):145-155.

[10]Dimitriadis SI, Salis C, Tarnanas I, Linden DE. Topological filtering of dynamic functional brain networks unfolds informative chronnectomics: A novel data-driven thresholding scheme based on orthogonal minimal spanning trees (OMSTs). Front Neuroinform. 2017;11:28.

[11]Barnett L, Seth AK. The MVGC multivariate Granger causality toolbox: a new approach to Granger-causal inference. J Neurosci Methods. 2014;223:50-68.

[12]Hassan M, Shamas M, Khalil M, El Falou W, Wendling F. EEGNET: An OpenSourceTool for Analyzing and Visualizing M/EEG Connectome. PLoS One. 2015;10(9):e0138297.

**Table 1: Comparison of the network organization between wakefulness and sleep stages**

|  | Median  Wakefulness  (P25-P75) | Median  REM  (P25-P75) | Median  Stage 3  (P25-P75) | Median  Stage 4  (P25-P75) | P-Value |
| --- | --- | --- | --- | --- | --- |
| **Male Sex**  **(n=28)** |  |  |  |  |  |
| ***Time domain*** |  |  |  |  |  |
| SWC | -0.002 (-0.005 – 0.014) | 0.054 (0.035 – 0.091) | 0.009 (0.0004 – 0.033) | 0.006 (-0.003 – 0.034) | <0.001^a,d,e^ |
| ***Frequency domain*** |  |  |  |  |  |
| SWC - power bands β | -0.002 (-0.004 – 0.004) | 0.075 (0.042 – 0.110) | 0.013 (-0.001 -0.043) | 0.003 (-0.007 -0.039) | <0.001^a,d,e^ |
| SWC - power bands σ | -0.006 (-0.011 – 0.008) | 0.068 (0.031 – 0.110) | 0.011 (-0.003 – 0.046) | 0.005 (-0.006 – 0.042) | <0.001^a,d,e^ |
| SWC - power bands α | -0.005 (-0.011 – 0.010) | 0.076 (0.042 – 0.109) | 0.002 (-0.004 – 0.028) | 0.004 (-0.006 – 0.044) | <0.001^a,d,e^ |
| SWC - power bands θ | -0.004 (-0.010 – 0.012) | 0.065 (0.032 – 0.120) | 0.011 (-0.007 – 0.035) | -0.001 (-0.007 – 0.028) | <0.001^a,d,e^ |
| SWC - power bands δ | -0.004 (-0.011 – 0.011) | 0.068 (0.049 – 0.103) | 0.013 (-0.004 – 0.061) | 0.002 (-0.005 – 0.036) | <0.001^a,d,e^ |
| **Woman Sex**  **(n=16)** |  |  |  |  |  |
| ***Time domain*** |  |  |  |  |  |
| SWC | 0.010 (0.002 – 0.035) | 0.094 (0.060 – 0.147) | 0.036 (0.007 – 0.061) | 0.022 (-0.004 – 0.044) | <0.001^a,d,e^ |
| ***Frequency domain*** |  |  |  |  |  |
| SWC - power bands β | -0.001 (-0.010 – 0.038) | 0.122 (0.083 – 0.174) | 0.042 (0.001 – 0.064) | 0.015 (-0.009 -0.045) | <0.001^a,d,e^ |
| SWC - power bands σ | -0.002 (-0.013 – 0.036) | 0.120 (0.084 – 0.156) | 0.028 (-0.006 – 0.065) | 0.019 (-0.009 – 0.052) | <0.001^a,d,e^ |
| SWC - power bands α | -0.005 (-0.020 – 0.043) | 0.133 (0.077 – 0.172) | 0.018 (-0.007 – 0.061) | 0.012 (-0.009 – 0.050) | <0.001^a,d,e^ |
| SWC - power bands θ | 0.008 (-0.009 – 0.033) | 0.115 (0.075 – 0.173) | 0.038 (-0.003 – 0.071) | 0.012 (-0.013 – 0.039) | <0.001^a,d,e^ |
| SWC - power bands δ | -0.003 (-0.014 – 0.033) | 0.124 (0.082 – 0.172) | 0.047 (0.010 – 0.071) | 0.020 (-0.010 – 0.047) | <0.001^a,d,e^ |
|  |  |  |  |  | Friedman test |

After Bonferroni correction: ^a^ Wakefulness vs REM p<0.05, ^b^ Wakefulness vs Stage 3 p<0.05, ^c^ Wakefulness vs Stage 4 p<0.05, ^d^ REM vs Stage 3 p<0.05, ^e^ REM vs Stage 4 p<0.05, ^f^ Stage 3 vs Stage 4 p<0.05.

REM = Rapid eye movement sleep.
